# Environmental and occupational respiratory diseases – 1050. Determination of immunoglobulin E in asthmatic workers with respiratory infections of Al-Baiji oil refinery in Iraq

**DOI:** 10.1186/1939-4551-6-S1-P49

**Published:** 2013-04-23

**Authors:** Mohemid Al-Jebouri, Ashwaq Al-Doori

**Affiliations:** 1 College of Medicine, Department of Microbiology, University of Tikrit, Iraq; 2 Department of Biology, College of Science,University of Tikrit, Iraq

## Background

Irritant-induced occupational asthma suspected to occur within few hours of exposure to high concentration of gas, fume or vapour at work in oil refinery establishmen lacking safety measures. There was a relationship between asthmatic and bacterial agents causing respiratory infections.

## Methods

The quatitative estimation of immunoglobulin E in serum of the patient was based on solid phase enzyme-linked immunosorbent assay (ELISA).

## Results

37% (74/200) of refinery patients and 19.5% (39/200) of nonrefinery hospitalized patients who had a total IgE level up to 100 IU/ml or more were considered as allergic. It was noticed that IgE level among refinery allergic patients ranged between 201-728 and 106-3266 IU/ml of refinery and nonrefinary hospitalized patients respectively.

## Conclusions

The highest incidence of allergy was recorded among refinery patients. The present study revealed that the percentages of 80 and 42 pathogenic strains were isolated from 74 and 39 allergic patients with respiratory infections from refinery and nonrefinery hospitalized patients respectively. The high incidence of asthma and respiratory infections in the oil refinery plant could be due observed lacking of safety measures.

